# Establishing Good Samaritan programs in healthcare institutions: a method for enhancing patient experiences and increasing loyalty

**DOI:** 10.1186/s12913-018-3677-6

**Published:** 2018-12-14

**Authors:** James K. Elrod, John L. Fortenberry

**Affiliations:** 1 Willis-Knighton Health System, 2600 Greenwood Road, Shreveport, LA 71103 USA; 2 LSU Shreveport, 1 University Place, Shreveport, LA 71115 USA

**Keywords:** Customer service, Patient relations, Patient experiences, Hospitals, Healthcare

## Abstract

**Background:**

Healthcare institutions deliver essential services that practically everyone needs at least periodically over the course of their lives. Hospital admissions, in particular, place patients in circumstances very foreign to them, with uncertain outcomes often being the case, causing stress for care recipients and their loved ones. Health and medical establishments must be very mindful of this and work toward supplying environments that are as comforting as possible for those in their care.

**Discussion:**

One avenue for relieving patients and their loved ones of hardships that complicate already difficult situations involves the development and implementation of Good Samaritan programs. Termed in reference to the biblical Good Samaritan who attended to the physical and emotional needs of his neighbor, these programs supply aid and comfort over and above that which traditionally has been delivered to patients in healthcare delivery settings, fostering satisfaction, positive word-of-mouth communications, and loyalty. Willis-Knighton Health System operates a Good Samaritan program, notably including a patient concierge team, complimentary lodging, and free transportation, with the offering greatly improving the patient experience. This article profiles Willis-Knighton Health System’s Good Samaritan program and supplies a blueprint for establishing similar offerings in virtually any healthcare entity.

**Conclusions:**

Good Samaritan programs provide significant support and assistance to care recipients and their loved ones at particularly difficult times. Willis-Knighton Health System has observed firsthand the many benefits afforded by its Good Samaritan program, relieving those served by the establishment of many burdens and endearing them to the institution for its associated investments which foster peace of mind.

## Background

Health and medical institutions deliver essential services that most everyone needs at least periodically over the course of their lives. Despite the dramatic advancements of modern medicine, injuries, illnesses, and other health-related concerns remain part of the human experience. Since these matters often cannot be addressed properly without the assistance of trained medical personnel, individuals must look to healthcare institutions for associated interventions and remedies, placing them in the role of patient, a position very much outside of the comfort zones of most [1–4].

Hospital admissions, in particular, can be highly worrisome for patients. Often enduring pain and facing, in some cases, very uncertain outcomes, patients must cope with significant stress stemming from their personal medical circumstances. When these strains are combined with the hardships associated with navigating through myriad processes and procedures in institutions which very often appear as complex labyrinths to the unfamiliar, the essence of the burdens patients must endure becomes quite clear [2, 5–7]. Such suffering usually doesn’t occur in isolation, either, as many patients are accompanied by loved ones who face equal frustration, stress, and bewilderment as they attend to their injured or ill friends and family members during various health system interactions. And on top of all of this, life—outside of given patient encounters—goes on, complete with all of its usual demands, further exacerbating already difficult situations [5, 8–10].

Healthcare experiences clearly have the potential to turn the worlds of patients and their loved ones upside down. Outstanding healthcare providers, of course, will deliver exceptional care and attention, covering the medical part of associated hardships quite well. But as indicated in the passage above, medical experiences often generate burdens which extend well beyond the realm of care and treatment. With this being the case, health and medical establishments which are truly conscientious will not limit themselves solely to addressing medical services; they’ll also direct attention toward remedying patient hardships falling outside of the boundaries of medical care, when and where possible [11, 12]. Indeed, the very best of healthcare providers will never lose sight of the totality of burdens faced by those in their care, striving to reduce or eliminate hardships at every available opportunity [13].

Realizing the benefit of value-added services that complement health and medical offerings and relieve undue stress on those seeking care, Willis-Knighton Health System instituted a special offering known as the Good Samaritan program. This program, termed in reference to the biblical Good Samaritan who attended to the physical and emotional needs of his neighbor, supplies aid and comfort over and above that which traditionally has been delivered to patients in healthcare delivery settings, fostering satisfaction, positive word-of-mouth communications, and loyalty. Willis-Knighton Health System’s Good Samaritan program notably includes a patient concierge team, complimentary lodging, and free transportation, yielding an array of services which greatly relieves burdens on patients and their loved ones [13]. This article profiles Willis-Knighton Health System’s Good Samaritan program and supplies a blueprint for establishing similar offerings in virtually any healthcare entity.

### Willis-Knighton Health System and its Good Samaritan program

Willis-Knighton Health System is a nongovernmental, not-for-profit healthcare provider delivering comprehensive health and wellness services through multiple hospitals, numerous general and specialty medical clinics, an all-inclusive retirement community, and more. Based in Shreveport, Louisiana, the system holds market leadership in its served region, centered in the heart of an area known as the Ark-La-Tex, where the states of Arkansas, Louisiana, and Texas converge. The system’s origins date to 1924 with the establishment of Tri-State Sanitarium, founded to address the healthcare needs of the burgeoning population of west Shreveport. Sold in 1929 to Drs. James Willis and Joseph Knighton, the establishment continued operations and, in 1952, it was renamed in honor of Drs. Willis and Knighton.

For the first several decades of its existence, the establishment played an important but relatively small role in delivering the region’s healthcare. But in the 1970s, having acquired a loyal following of patients in the west Shreveport marketplace, executives made the decision to expand into neighboring markets in an effort to touch more lives. In doing so, Willis-Knighton Health System turned to various structure, product, and process innovations, adopting the hub-and-spoke model of organization design [14, 15], establishing centers of excellence [16], and embracing the practice of adaptive reuse [17, 18], with each of these approaches notably emerging from outside of the healthcare industry [19]. In advancing the institution’s capacity to serve, however, executives wanted to ensure that the human side of healthcare delivery was not lost. This prompted the search for an innovative approach to customer service, something especially important as Willis-Knighton Health System’s growth pursuits would lead it into markets dominated by larger, more powerful competitors [13]. Executives knew that capturing market share in such environments would be difficult or impossible if it merely matched the customer service initiatives of rivals. Instead, a new and innovative approach to addressing the wants and needs of patients would be required, helping growth-aspiring Willis-Knighton Health System to leverage interest and attention, setting the stage for increased patronage.

In seeking to identify a unique approach, executives first investigated the customer service initiatives of current and anticipated rivals in the greater Shreveport marketplace, discovering that every activity embraced by competitors centered on matters of health and medical care. This was to be expected, of course, as each entity was in the business of healthcare delivery. But on deeper inspection, Willis-Knighton Health System realized that this usual and customary approach to customer service in the healthcare industry failed to take into consideration the totality of burdens faced by patients, revealing a potential avenue for achieving a critical point of differentiation that could benefit care recipients, supply competitive advantages, and bolster growth. The search then advanced, seeking to identify complementary services with the potential to differentiate Willis-Knighton Health System from other institutions. This prompted the institution to look outside of the healthcare industry for a solution that could be adopted for use within, something which the institution was growing increasingly adept at doing [13, 19].

Reflecting deeply on the patient experience, executives developed a generic profile of issues faced by patients in an effort to identify a parallel customer from another industry, permitting possible insights into service differentiators that could be transferred into a healthcare delivery setting. Generically described, patients are away from home in an unfamiliar environment, they have many questions about their new environment and its successful navigation, they often are accompanied by family and friends, and the experience is not an end unto itself, but instead, it is a means to a desired end. This scenario very much parallels that experienced by a traveler during a hotel stay, leading executives to investigate how the hotel industry addressed its patrons. Quality hotels had long focused on the totality of the circumstances faced by travelers, notably supplying things such as complimentary transportation to and from airports and other travel hubs and providing all-important and enormously helpful concierge services. These value-added services stretched well beyond the core product offering of supplying overnight accommodations for guests. Of course, such services were not supplied by all hotels; they were offered just by the better ones, and they served as differentiators, distinguishing quality establishments from their competitors.

On reflection, Willis-Knighton Health System saw no reason why it could not follow the example provided by quality hotels, offering similar distinctive, ancillary services to complement core health and medical offerings. After deeply examining what would be most appealing to its clientele in the context of the institution’s abilities, a trio of value-added services emerged, affording patients and their loved ones access to a patient concierge team, complimentary lodging, and free transportation. Since these services were designed to provide support for those facing what very often are distressing circumstances and situations, Willis-Knighton Health System decided to package the array into an offering known as the Good Samaritan program [13].

### Service profiles

Since its establishment, Willis-Knighton Health System’s Good Samaritan program has provided relief to patients and their loved ones from burdens often faced as they go about addressing matters of illness and injury. To better understand the depth and breadth of value-added services offered by this program, brief depictions of the noted array are provided as follows.

#### Patient concierge services

The core component of Willis-Knighton Health System’s Good Samaritan program centers on its patient concierge services, delivered through patient representatives operating at each of the institution’s establishments. These representatives, each reporting directly to the given facility’s top administrative officer, perform in much the same capacity as concierge staff members do at quality hotels. Among other things, they greet patients and their loved ones as they enter the hospital, ensure that they are directed properly to their given destinations within the establishment, and offer helpful assistance, as needed, with their goal being to foster customer service and support at every possible opportunity. Patient representatives serve as the gateway to value-added services provided through Willis-Knighton Health System’s Good Samaritan program, circulating as needed throughout assigned institutions. They offer far more than that provided by the typical customer service agents that are now fairly common in healthcare institutions, as the following examples illustrate.

- A patient admitted to Willis-Knighton Medical Center through the hospital’s emergency department left her home rather hurriedly as a result of her emergent medical condition, preventing her from attending to things before leaving, with the most concerning one being arranging for the care of her kittens. She very obviously was worried about them and she communicated her concerns to Willis-Knighton Health System’s patient representative. Shortly thereafter, the patient representative departed the institution and visited the patient’s household where she fed the kittens and arranged for a neighbor to care for them until the patient was released from the hospital. On learning that her kittens were fed and in good hands, the patient was highly relieved and was able to concentrate fully on her recovery [13].
- A patient admitted to WK Pierremont Health Center refused to eat any of the food presented before her at the hospital. Despite a wide range of efforts, staff members were unable to convince the patient to eat, something that was especially important for restoration of health and eventual release from the hospital. Willis-Knighton Health System’s patient representative met with the patient and learned that she liked a dish served at a particular restaurant in town located a short distance from the hospital. After checking with the attending physician to ensure that consuming such a meal would not cause any negative health effects, Willis-Knighton Health System’s patient representative visited the restaurant and ordered the favorite dish, requesting it as a carry-out order. On return to the hospital and presentation of the order, the patient eagerly and happily consumed the meal, setting the stage for her recovery and discharge from care.
- A patient, accompanied by her husband, was admitted to Willis-Knighton Medical Center with failing health, necessitating intensive care and treatment. Her meals were supplied as part of her admission; her husband’s meals, of course, were not. Being away from home in another city, options were limited due to the couple’s poverty; they had no money for food. On learning of this, Willis-Knighton Health System’s patient representative consulted with the hospital’s administrator and arranged for the patient’s husband to receive meals free of charge from the hospital’s cafeteria, greatly relieving his stress and permitting him to focus fully on his wife’s care and treatment.
- On Willis-Knighton Health System’s establishment of an indigent clinic in one of Shreveport’s poverty-stricken neighborhoods, staff members were perplexed as to why more members of the community were not scheduling appointments to receive the free medical services newly available in the area. It was discovered that many were reluctant to leave their homes fearing that their residences would be burglarized while they were away, given the high crime in the community. On learning of this, steps were taken to dispatch patient representatives to house-sit while the homeowners received care at the clinic. This supplied an interim solution while executives sought a better remedy which ultimately arrived through improved patrols by local law enforcement, courtesy of funding provided by Willis-Knighton Health System’s Tithing the Bottom Line program, which takes a portion of the health system’s earnings and directs these resources to fund pursuits that improve quality of life in the community. This investment bolstered law enforcement initiatives in the area and helped the public regain confidence in the integrity of their neighborhood. As order was being restored through these new initiatives, patient representatives filled a critical gap which permitted many to attend to their healthcare needs without worrying about their households.

These depictions represent just a few of scores of examples which indicate the lengths to which patient representatives will go to assist those in need. These interventions very often have little to nothing to do with medical care and treatment, but they all have direct effects and impacts on given care experiences, courtesy of the removal of burdens experienced by patients and their loved ones, permitting them to concentrate their attention on matters of health.

#### Complimentary lodging

Willis-Knighton Health System’s complimentary lodging service is perhaps the most highly evolved offering of its Good Samaritan program, having been developed decades ago, with continual advancements being incorporated over time in tandem with the institution’s expanding footprint. Friends and family members who attend to the needs of loved ones during hospital admissions offer sincere comforts, but such dedication carries its own set of hardships, as they must endure lengthy expanses of time in public waiting areas between brief visits with their care-receiving loved ones. Such inconveniences are compounded for those residing out-of-town, as this necessitates either extensive commutes or overnight accommodations in a hotel; things that can prove very costly, especially for lengthy admissions [13]. Further, with Willis-Knighton Health System’s historical focus on serving the underserved [13, 18, 20], some could even be prohibited from attending to the needs of their loved ones altogether, given limited access to personal transportation or the funding required for overnight accommodations. Willis-Knighton Health System viewed such hardships to warrant an associated intervention through its Good Samaritan program, leading the institution to offer complimentary lodging services.

The core of Willis-Knighton Health System’s complimentary lodging offering rests in each of its hospitals where unoccupied patient rooms are made available to the loved ones of admitted patients, with priority being given to the friends and family members of those in intensive care, those from out-of-town, and those needing opportunities for respite. Booking rooms is handled by patient representatives and, if after normal business hours, by hospital switchboard operators. On-duty nursing supervisors monitor guests and determine priority of use when vacancy is limited. As long as unoccupied patient rooms remain available, no one in need of accommodations will be denied. While this particular amenity costs very little to provide, requiring nothing more than light housekeeping, laundry services, and minor coordination by staff members, the benefits are monumental. The accommodations obviously help the loved ones of patients, but even the patients themselves benefit through the peace of mind of knowing that their friends and family members are nearby, safe, and comfortable [13].

In recent years, Willis-Knighton Health System has enhanced its lodging program by establishing two additional options which are priced according to ability to pay, ranging from approximately one-half of the cost of an area hotel room to completely free of charge. The first option is located at The Oaks of Louisiana, the institution’s senior living community, where a special group of apartments has been designated to accommodate patients and their loved ones who reside out-of-town and are facing lengthy admissions. The second is located at The Arbors Apartments, an institution-owned apartment complex situated adjacent to WK South and its Center for Women’s and Children’s Health, which serves primarily to accommodate women encountering difficult pregnancies, sick infants and children, and their associated loved ones. Given the robust lodging resources developed by Willis-Knighton Health System, overnight accommodations for served audiences in need can almost always be provided.

#### Free transportation

In the greater Shreveport region, public transportation is very limited or completely unavailable, placing hardships on many as they seek to address their healthcare wants and needs [13, 18]. Willis-Knighton Health System has taken steps to reduce such hardships by strategically placing its facilities across its served market, minimizing travel distances from virtually any point in the region, fostering expeditious access to care [14, 15]. Even so, if one does not have access to personal transportation or is too ill to drive safely, the timely receipt of care can be hampered [21–23]. For this reason, Willis-Knighton Health System introduced a complimentary transportation service, offering patients with non-emergent medical conditions the opportunity to arrange for transit between and among any Willis-Knighton Health System facility, including its rural network partners [15].

Complementing the institution’s highly-robust emergency transportation network which includes both ground and air ambulances [14], Willis-Knighton Health System’s non-emergent transportation network closed remaining transportation gaps, removing a common concern of some patients that they might not be able to “get a ride” to their appointment, examination, or admission. This service also supports Willis-Knighton Health System’s operation as a hub-and-spoke network which, by design, requires an excellent transportation infrastructure to ensure expeditious access from spoke facilities which provide more limited service arrays to the hub facility which offers a full service array, including the most intensive medical interventions available within the system [14, 15]. A fleet of shuttle buses, patient transit vans, and automobiles is used for transporting patients, with vehicle selection being determined by the needs of those requiring transit services. Costs associated with provision of this offering are surprisingly low as the amenity essentially extends the deployment of existing resources. This particular component of Willis-Knighton Health System’s Good Samaritan program facilitates access to care, eliminates transportation expenditures which otherwise would be required of patients, and dramatically enhances personal convenience.

### Operationalization

**Fig. 1:**
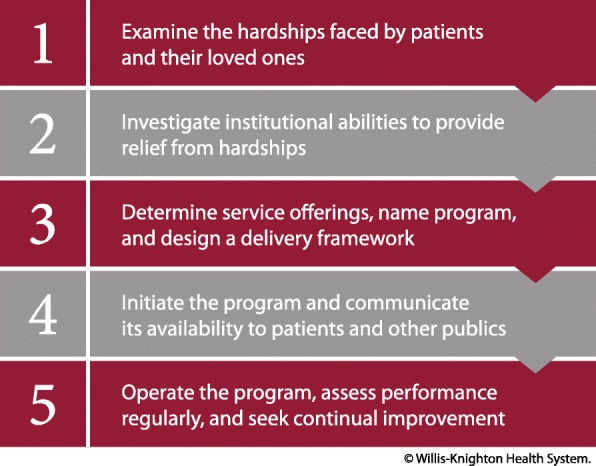
Steps for establishing a Good Samaritan program

Willis-Knighton Health System’s Good Samaritan program has supplied numerous mutual benefits, providing patients and their loved ones with value-added services and offering the institution a competitive advantage that has enhanced patient satisfaction and loyalty. While novel at the time of its introduction, today, some healthcare providers have devised similar programs in their efforts to deliver and capture associated benefits. Many more, however, remain focused solely on matters of health and medical care, without providing the supplementary benefits that fill one or more needs falling beyond the realm of treatment. Of these institutions, at least some might be desirous of establishing such programs, with the following steps, outlined in Fig. 1, supplying a systematic framework for doing so.

#### Step 1: examine the hardships faced by patients and their loved ones

Arguably, the most important step in establishing a Good Samaritan program entails the careful examination of hardships faced by patients and their loved ones. Due to variations in service type, focus, setting, location, and other variables differentiating healthcare institutions, the hardships encountered by given audiences can vary greatly, requiring each institution to inquire directly with their patient clientele for insights and ideas on associated burdens. Accuracy here is important, as healthcare institutions want to ensure that potential remedies offered indeed address challenges faced by clients, improving the overall patient experience.

#### Step 2: investigate institutional abilities to provide relief from hardships

The challenges faced by patients and their friends and family members which fall outside of the realm of healthcare delivery likely will be numerous and highly varied, given life’s complexities. Providing comprehensive relief, of course, is not feasible. Instead, healthcare institutions should focus on common hardships experienced, and for each of these, investigate possible avenues of relief which might be offered. Remedies may or may not be possible for some hardships faced, but others perhaps can be addressed. Creative thinking is very helpful during this particular step. Healthcare institutions might even consider establishing partnerships with external companies for the provision of critical, value-added services. Suppose, for example, that a hospital which noted patient transportation hardships did not have the resources, expertise, or desire to initiate an in-house transportation operation. Instead of forgoing the offering altogether, the hospital might possibly contract with an area taxi company or other transportation provider to ensure that transit hardships faced by clients do not impede access to care. Whether supplied in-house or outsourced, the relief provided will be most welcomed by patients and their loved ones, fostering goodwill and loyalty.

#### Step 3: determine service offerings, name program, and design a delivery framework

On careful consideration of the hardships experienced by patients and their loved ones in the context of institutional abilities to address associated challenges, healthcare establishments must determine the value-added services which they will provide. On doing so, the array should be compartmentalized into a program, named as desired in a manner reflective of the services offered. Even if a single service is provided, placement of such into a formal program will set the stage for expanding the depth and breadth of services when warranted. This also is especially helpful for publicizing the program. A delivery framework should be developed, noting responsible parties and reporting relationships, indicating protocols for service provision, and depicting other policies of organization and operation. A blueprint for program delivery should emerge by the conclusion of this particular step.

#### Step 4: initiate the program and communicate its availability to patients and other publics

Once the program has been developed fully, it is to be operationalized, accordingly, with the given healthcare entity taking steps to ensure its active communication to patients and other publics of the institution. Both formal and informal communication channels can be used for this, with the goal being to ensure that the program is well publicized. Given the numerous mutual benefits supplied by these programs, intensive communication efforts are encouraged.

#### Step 5: operate the program, assess performance regularly, and seek continual improvement

With steps to actively communicate the offering underway, program operations begin in earnest, with care being taken to monitor performance to ensure delivery of value as intended to patients and their loved ones. Importantly, opportunities to continually improve the program should be sought, either by enhancing existing services or adding new ones. Such improvements obviously will benefit patients and their loved ones, but they also will benefit the institutions providing such. Since these programs serve to differentiate institutions, providing associated competitive advantages, it should be expected that rivals will endeavor to offer similar programs soon after implementation. By continually developing the program, given institutions can stay one step ahead of those replicating offerings, creating an enduring competitive advantage. By directing attention in the present toward the potential product—that which the offering might become in the future [12, 24, 25]—healthcare institutions will have a blueprint for enhancing their programs over time; something that will benefit clients and establishments alike.

## Conclusions

Through provision of unique offerings which supplement the health and medical services of healthcare institutions, Good Samaritan programs provide significant benefits to patients and their loved ones, reducing at least some of the burdens experienced as they go about addressing associated health concerns. The relief afforded by these programs permits the attention of patients and their friends and family members to be directed toward matters of healthcare, allowing concentrated efforts to center on resolving illness and injury. Provision of such offerings greatly advances the customer service initiatives of healthcare institutions, improving patient satisfaction, hastening positive word-of-mouth communications, and building associated loyalty, all factors with the potential to enhance institutional viability and vitality.

Willis-Knighton Health System’s Good Samaritan program was introduced decades ago and continues to deliver exceptional, mutually beneficial value to this very day. It has become a core component of the organization’s culture, serving as a continual reminder to all staff members of the value of expressing concern for the complete patient. This particular focus—the complete patient—is often spoken about in healthcare institutions, but in order for value to be derived, good intentions must be complemented by tangible actions, with Good Samaritan programs serving as the ideal vehicle for such. Opportunities abound for like programs to be offered by healthcare institutions of all types and sizes. Even a modest value-added service which supplements excellent medical care will be welcomed by clients of given healthcare institutions, making program operation possible for most any healthcare provider. Excellent patient experiences and valued institutional outcomes await.
